# Anti-HBs persistence and anamnestic response among medical interns vaccinated in infancy

**DOI:** 10.1038/s41598-025-00055-w

**Published:** 2025-05-09

**Authors:** Nesrine Fathi Hanafi, Nashwa Naguib Omar, Ghada Abdelwahed Ismail, Amani Ali El-Kholy, Ahmed ElShafei, Sara AbdelAziz Essa

**Affiliations:** 1 Department of Medical Microbiology and Immunology, Faculty of Medicine, Alexandria University, Alexandria, Egypt; 2 Department of Clinical Pathology, Faculty of Medicine, Ain Shams University, Cairo, Egypt; 3 Department of Clinical Pathology, Faculty of Medicine, Cairo University, Cairo, Egypt; 4 Modern University for Information and Technology, Cairo, Egypt

**Keywords:** Hepatitis B, Vaccine, Immunization, Booster, Egypt, Microbiology, Gastroenterology, Health care, Health occupations

## Abstract

Medical interns are at high risk of acquiring Hepatitis B Virus (HBV) infection during their training. HBV vaccination is the most effective measure to reduce the global incidence of HBV. The duration of protection after HBV vaccination is still controversial. We aimed to determine the prevalence of protective anti-HBs levels among medical interns who had received compulsory hepatitis B vaccination in infancy, and to assess the anamnestic response of those subjects with non-protective antibodies titers, to a booster dose of the vaccine. We conducted a cross-sectional study on 519 medical interns in 2022. We examined their immunization status and records. Blood samples were collected and qualitative testing of Hepatitis B surface antigen (HBsAg), and quantitative testing of Hepatitis B surface antibody (anti-HBs) were performed. For medical interns whose titers were ˂ 10 mIU/mL, a booster dose of the vaccine was given, followed by repeat testing of anti-HBs 2 months later. About 304 (58.6%) of the medical interns revealed titers higher than or equal 10 mIU/mL. About (44.93%) of male medical interns showed an immunity level below 10 mIU/mL. However, (43.91%) of female medical interns had an antibody titer of 100 mIU/mL or higher. All subjects who got a booster dose presented a titer level of 10 mIU/mL 2 months later. About (41.4%) of our medical interns enrolled in the study had anti-HBs titer ˂ 10 mIU/mL. This raises the importance of establishing a screening protocol and offering booster dose for those at risk to protect them against the high risk of infection.

## Introduction

Hepatitis B virus (HBV) is a significant global health issue. The World Health Organization (WHO) Western Pacific Region and WHO African Region have the largest infection burdens, with 97 million and 65 million chronically infected persons, respectively. Moreover, it is estimated that approximately 59 million healthcare workers are exposed to a variety of occupational hazards daily, the most common of which is the risk of contact with infected patients and/or infected materials, such as body fluids, contaminated medical supplies, equipment, and environmental surfaces^1^.

Hepatitis B virus is a significant pathogen in acute and chronic hepatitis, and it can lead to cirrhosis and hepatocellular cancer^2^. Hepatitis B virus (HBV) is considered the most transmissible blood-borne virus following percutaneous exposure and contact with infected blood and body fluids among healthcare workers. This is the foundation for recognizing HBV as a significant occupational danger for HCWs and medical interns in training all around the world, particularly those in highly endemic zones^3,4^.

A recent systematic review with meta-analysis in Egypt, analyzing research published between 2000 and 2022, found that children under 20 who had received HBV vaccination during infancy had the lowest prevalence rate of 0.69%^5^. Yet, the national pooled prevalence of HBV in Egypt remains unknown.

Vaccination is the most effective measure to reduce the global incidence of HBV. Safe and effective recombinant vaccines against hepatitis B have been available since 1986. The vaccine has numerous indications, in addition to all infants and any unvaccinated children, the Advisory Committee on Immunization Practices recommends primarily vaccinating any adults who may have a higher risk for contracting or complication from hepatitis B as healthcare workers, dialysis patients and persons at risk for sexually transmitted disease^6^.

Egypt has routinely conducted vaccinations against Hepatitis B as part of its immunity program since 1992. HBVaccine (r DNA) serum Institute of India Pvt. Ltd, it consists of 1 monovalent birth dose followed by 2 doses of hepatitis B-containing combination vaccine administered during the same visits as the first and third doses of DTP-containing vaccines^7^. A primary 3-dose series induces protective antibody concentrations in > 95% of healthy infants, children, and young adults^8,9^. Strong immune memory is necessary for the long-term protection provided by HB immunization. Numerous investigations aim to assess T-cell memory specific to HBsAg, especially in recipients of vaccines whose serum anti-HBs level is not very protective, to establish the most suitable course of action for booster vaccinations^10,11^.

Research currently available in the literature indicates that immunological memory endures even if the anti-HBs titer falls below 10 mIU/mL, as demonstrated by an amnestic response following the administration of an HBV booster dose^12^.

Nevertheless, screening to establish the extent of vaccine-induced immune response is limited in Egypt, as in most low-and-middle-income countries. Medical interns and residents are at high risk of HBV infection through occupational exposure to blood and other body fluids during their clinical training in hospitals and medical facilities. Ideally, this group should be adequately assessed to ensure immunity against HBV infection, however, only a few publications are available on the sustained immunity of medical interns against HBV after vaccination in infancy and childhood.

This study aimed to determine the prevalence of protective anti-HBs levels among medical interns who had received compulsory hepatitis B vaccination in infancy and to assess the anamnestic response of those subjects with non-protective antibody titers, after providing a booster dose of the vaccine.

## Methods

### Study design

A descriptive cross-sectional study was conducted at Alexandria Main University Hospital.

Inclusion criteria:

- Medical intern who received the full compulsory hepatitis B vaccine series in infancy, from their primary healthcare records.
- Medical intern HBsAg negative.

Exclusion criteria:

- Any medical intern who cannot guarantee receiving the full compulsory hepatitis B vaccine series in infancy.
- Any medical intern who received an additional vaccination series, or booster dose after the full compulsory hepatitis B vaccine series in infancy.
- Any medical intern known to be non-responsive to hepatitis B vaccine.

### Data collection method

A secondary source of data from the Infection Prevention and Control Unit registry at Alexandria Main University Hospital during their immunization campaign was used. A comprehensive sample of 531 medical interns were eligible for the quantitative determination of anti-HBs titers, between October 2022 and November 2022.

### Venous blood sampling

A venous blood sample (3–5 mL) was withdrawn from each subject aseptically. The serum was separated by centrifugation, aliquoted into two labeled sterile Eppendorf tubes, and stored at − 70 °C till processing.

### Detection of HBsAg

All serum samples were tested for HBsAg to exclude HBsAg positivity. Qualitative detection of HBsAg was carried out using (Dialab^®^ Double antibody sandwich ELISA for the cut-off determination of HBsAg in human serum or plasma, Austria).

### Detection and quantification of anti-HBs titer

Specimens submitted for detection of anti-HBs using a chemiluminescent microparticle immunoassay (CMIA). The test is indicated for assessing the response to HBV vaccination. An anti-HBs antibody concentration of more than or equal 10 mIU/mL was considered a reliable serological marker of long-term protection against HBV infection. For medical interns whose titers were less than 10 mIU/mL, a booster dose of the monovalent HBVaccine (r DNA) serum Institute of India Pvt. Ltd vaccine was provided, followed by repeat testing of anti-HBs 2 months later.

### Statistical analysis of the data

Data were fed to the computer and analyzed using IBM SPSS software package version 23.0. Descriptive presentation was done by percentage and range. Assessments between the different groups were evaluated using the Chi-square test. A value of *P* < 0.05 was considered statistically significant.

## Results

**Fig. 1:**
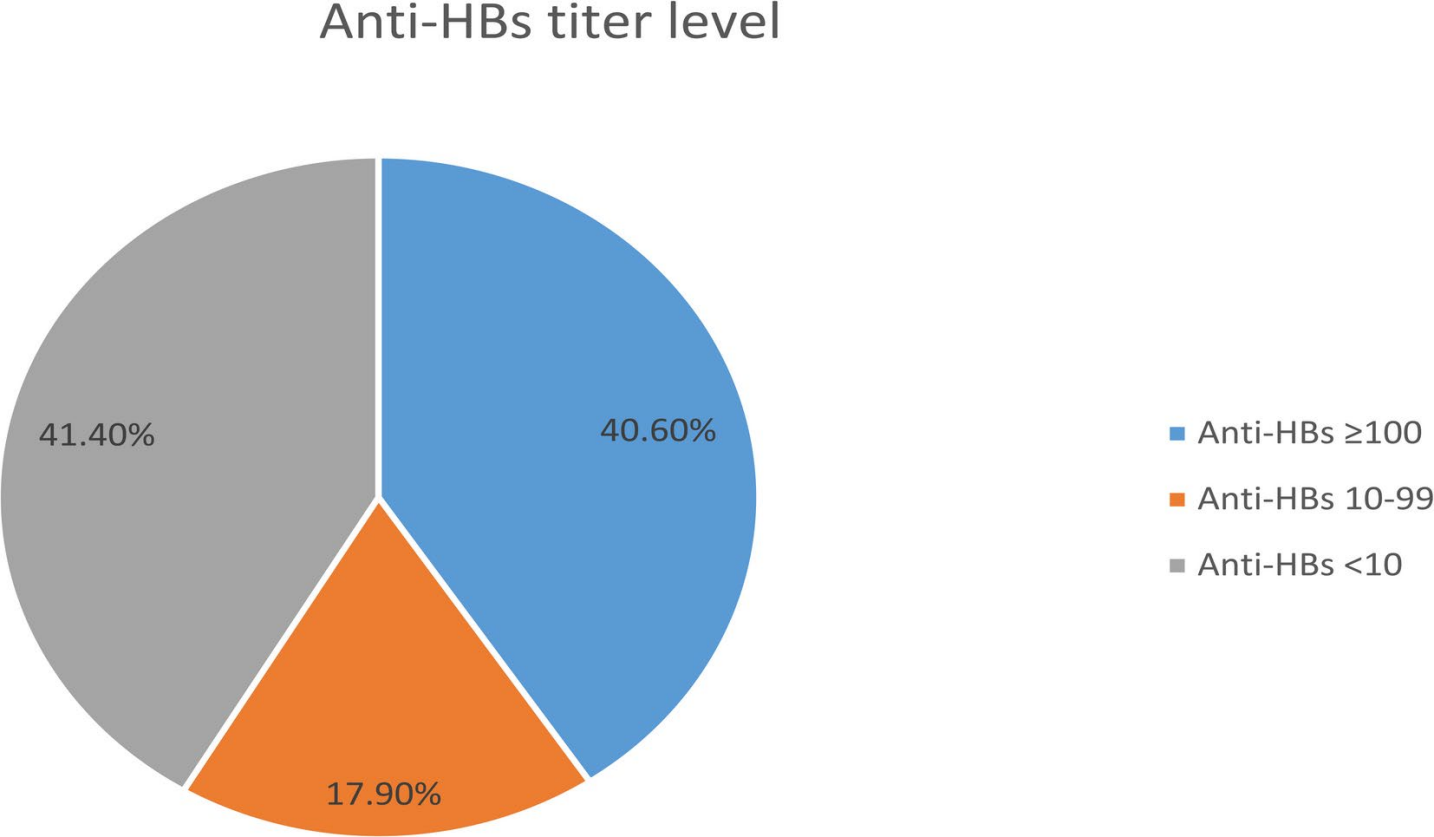
Distribution of anti-HBs titer among medical interns in Alexandria Main University Hospital, October 2022.

A total of 519 medical interns were enrolled in the study with a dropout of only (2.26%). Males represented (39.9% *n* = 207) of the studied group. The age ranged from 24 to 27 years old with a mean and mode of 25 years old. About 304 (58.6%) of the medical interns were revealed to have continuous immunity against hepatitis B (Fig. 1).

For all candidates an anti-HBs level > 10 strongly indicates immunity, but an anti-HBs level ˂ 10 does not necessarily mean someone is not immune. For all 215 candidates with immunity levels below to 10 mIU/mL, two months after a booster dose the antibody titer raised above 10 mIU/mL (*P* < 0.0001).

Concerning gender, more than half of the enrolled male medical interns (55%) were found to have persistent immunity since the compulsory immunization series. Yet, the largest subcategory among males was those with antibodies below 10 mIU/mL. On the other hand, the largest female subcategory fell in the group with antibody levels equal to or more than 100 mIU/mL. No statistical difference was found among the different immunity levels with gender (Table 1).

**Table 1:** Classification of medical intern Anti-HBs titers at Alexandria main university hospital in relation to gender, October 2022.

Anti HBs level grades	Female (*n* = 312)	Male (*n* = 207)	Total no (519)
˂ 10 mIU/mL	122 (39%)	93 (45%)	215 (41.4%)
10–99 mIU/mL	53 (17%)	40 (19.3%)	93 (17.9%)
≥ 100 mIU/mL	137 (44%)	74 (35.7%)	211 (40.6%)
χ^2^	3.4732		
P	0.179		

## Discussion

Throughout their practical training, medical interns are surely professionally exposed to the risk of contracting infectious diseases, including HBV infection^13^. Several studies conducted on comparable populations have shown that people who are immunized at birth retain a protective antibody titer for several years thereafter, although the proportion decreases twenty years after the first immunization course^14–19^. Our research has demonstrated that the protective anti-HBs titer following childhood HBV vaccinations diminishes with age, as (41.4%) of the medical interns in the study had anti-HBs titer ˂ 10 mIU/mL. This raised the attention of necessary preventive action towards HCWs, and specially the interns before starting their practice at healthcare facilities. This is in accordance with Lamberti et al. about Italian medical students^20^. Similarly, Grosso et al. found that seroprotection persisted in 57.9% of HCWs who received vaccination in infancy^4^. Annisa et al. also found that 81% of the medical students enrolled in their study, who had been vaccinated in infancy, lacked a protective anti-HBs level and were at risk of HBV transmission while performing medical care^21^.

The duration of protection following HBV vaccination remains a topic of debate. The fact that immunity relies solely on anti-HBs antibodies is not supported by scientific evidence^22^. Since anti-HBs play a more significant role in the anamnestic response of cellular immunity. It was reported that persons without this seroprotective level of anti-HBs antibody (titres of < 10 mIU/mL) responded with a rapid rise in titer after a booster dose, indicating immunologic memory and persistence of protection^23^. Additionally, the European Consensus Group on Hepatitis B immunity stated that long-term protection with HBV vaccine may last for at least 15 years among immunocompetent individuals, however, a booster shot is advised when the titer falls below 10 mIU/mL^24^.

The current study showed no statistically significant variation based on gender. This was in line with the findings of a couple of studies^25–27^. Nonetheless, some research revealed a variation in anti-HBs titer dependent on gender. Pavani and his colleagues as well as Zamani et al. found that female healthcare workers had considerably greater levels of anti-HBs. They speculated that smoking and other traits associated with men would be likely causes^28,29^. Conversely, a national community-based cross-sectional study conducted in Egypt included 3600 completely vaccinated children between the ages of 9 months and 16 years, revealed that female gender was found to be a significant predictor of having a non-seroprotection level^19^.

It is noteworthy that in the present study, all participants who received a booster dose achieved anti-HBs titers exceeding 10 mIU/mL. This indicates that while anti-HBs levels in vaccine responders who have completed the vaccination series may decline over time, they remain immune and will mount a protective response when exposed to HBV^30,31^. This is consistent with the findings of Zhao et al., who found that following a booster dose, (93.1%) of the participants with anti-HBs antibodies < 10 mIU/mL had raised their immunity status^32^.

### Limitations of the study

The likelihood of generalizing the results was reduced by depending solely on the data that was provided and the medical intern’s voluntary involvement. Asymptomatic breakthrough HBV infection among apparently immune interns was not evaluated. Furthermore, insufficient detailed intern behaviors’ and demographic information were available to go deeper into the research groups.

## Conclusions

This study provides valuable insights into the persistence of anti-HBV immunity in individuals approximately two decades after early childhood vaccination. Our findings reveal that a significant percentage of medical interns, vaccinated at birth, exhibit non-protective anti-HBs titers at the time of their first employment. Therefore anti-HBs and HBsAg screening tests should be done to all medical interns before making any risky contact and also offering a booster dose to those non-immune to preserve immunity over time if necessary.

## Data Availability

Data is provided within the manuscript or supplementary information files.

## Declarations

### Recommendations

More date regarding specialty and history of sharp injuries would be considered in further researches that may be guided by this work.

## References

[1] World Health Organization (WHO). Occupational Health, Health Workers and Health Worker Occupational Health. Available online: http://www.who.int/occupational_health/topics/hcworkers/en/

[2] Pisano, M. B. et al. Viral hepatitis update: Progress and perspectives. *World J. Gastroenterol.* **14** (27(26), 4018–4044 (2021). 10.3748/wjg.v27.i26.4018 PMC8311538 34326611

[3] Lewis, J. D., Enfield, K. B. & Sifri, C. D. Hepatitis B in healthcare workers: Transmission events and guidance for management. *World J. Hepatol.* **7**, 488–497 (2015). 25848472 10.4254/wjh.v7.i3.488 PMC4381171

[4] Grosso, G. et al. Long-term persistence of seroprotection by hepatitis B vaccination in healthcare workers of Southern Italy. *Hepat. Mon*. **12** (9), e6025 (2012). 23087756 10.5812/hepatmon.6025 PMC3475028

[5] Azzam, A. et al. Seroprevalence of hepatitis B virus surface antigen (HBsAg) in Egypt (2000–2022): A systematic review with meta-analysis. *BMC Infect. Dis.* **10** (23(1), 151 (2023). 10.1186/s12879-023-08110-5 PMC10007808 36899311

[6] Advisory Committee on Immunization Practices; Centers for Disease Control and Prevention (CDC). Immunization of healthcare personnel: Recommendations of the advisory committee on immunization practices (ACIP). *MMWR Recomm Rep.* **60**, 1–45 (2011). 8668119

[7] WHO prequalified vaccines. December (2023). Available at https://extranet.who.int/prequal/vaccines/list-prequalified-vaccines accessed on 14.

[8] World Health Organization (WHO). Immunization coverage facts (2023). Available from URL: https://www.who.int/news-room/fact-sheets/detail/immunization-coverage accessed on 14 December 2023.

[9] Chen, D. S. Hepatitis B vaccination: The key towards elimination and eradication of hepatitis B.* J. Hepatol.*** 50**(4), 805–816 (2009). 10.1016/j.jhep.2009.01.002 19231008

[10] Spradling, P., Xing, J., Williams, R., Faleafaga, Y. & Dulski, T. Immunity to hepatitis B virus (HBV) infection two decades after implementation of universal infant HBV vaccination: Association of detectable residual antibodies and response to a single HBV challenge dose. *Clin. Vaccine Immunol.* **20** (4), 559–561 (2013). 23408522 10.1128/CVI.00694-12 PMC3623410

[11] Saffar, H., Saffar, M., Ajami, A., Khalilian, A. & Esfandabad, K. Long-term T-cell-mediated Immunologic memory to hepatitis B vaccine in young adults following neonatal vaccination. *Hepat. Mon*. **14** (9), e22223 (2014). 25368659 10.5812/hepatmon.22223 PMC4214124

[12] European Consensus Group on Hepatitis B Immunity. Are booster immunisations needed for lifelong hepatitis B immunity?. *Lancet* **355**, 561–565 (2000). 10683019

[13] La Torre, G. et al. Knowledge, attitude and behaviours towards recommended vaccinations among healthcare workers. *Healthcare*** 5**, 13 (2017). 10.3390/healthcare5010013 PMC5371919 28272332

[14] Romanò, L. et al. Persistence of immunity 18–19 years after vaccination against hepatitis B in 2 cohorts of vaccinees primed as infants or as adolescents in Italy. *Hum. Vaccin. Immunother*. **13**, 981–985 (2017). 28272974 10.1080/21645515.2017.1264795 PMC5443392

[15] Anutebeh, E. N. et al. Immune response to hepatitis B vaccine following complete immunization of children attending two regional hospitals in the Southwest region of Cameroon: A cross-sectional study. *BMC Infect. Dis.* **2** (21(1), 1205 (2021). 10.1186/s12879-021-06913-y PMC8641163 34856942

[16] Faustini, A. et al. Persistence of anti-HBs 5 years after the introduction of routine infant and adolescent vaccination in Italy. *Vaccine* **19** (20–22), 2812–2818 (2001). 11282191 10.1016/s0264-410x(01)00005-6

[17] Bagheri-Jamebozorgi, M. et al. The persistence of anti-HBs antibody and anamnestic response 20 years after primary vaccination with Recombinant hepatitis B vaccine at infancy. *Hum. Vaccin. Immunother*. **10** (12), 3731–3736 (2014). 25483689 10.4161/hv.34393 PMC4514033

[18] Stefanati, A. et al. Long-term persistency of hepatitis B immunity: An observational cross-sectional study on medical students and resident Doctors. *J. Prev. Med. Hyg.* **30** (60(3), E184–E190 (2019). 10.15167/2421-4248/jpmh2019.60.3.1315 PMC6797890 31650052

[19] Salama, I. I. et al. Effectiveness of hepatitis B virus vaccination program in Egypt: Multicenter National project. *World J. Hepatol.* **7** (22), 2418–2426 (2015). 26464758 10.4254/wjh.v7.i22.2418 PMC4598613

[20] Lamberti, M. et al. Vaccination against hepatitis B virus: Are Italian medical students sufficiently protected after the public vaccination program? *J. Occup. Med. Toxicol.* **10**, 41 (2015). 26539242 10.1186/s12995-015-0083-4 PMC4632277

[21] Annisa, Zain, L. H. & Ricke, L. Protection status against hepatitis B infection assessed from anti-HBs level, history of vaccination and history of infection based on anti-HBc in medical students.* IOP Conf. Ser. Earth Environ. Sci.*** 125**, 012104. 10.1088/1755-1315/125/1/012104 (2018).

[22] Hofmann, F. & Kralj, N. Criteria for successful hepatitis B vaccination in adults: Results of a case study. *Infection* **37** (3), 266–269 (2009). 18854934 10.1007/s15010-008-7410-y

[23] Publication, W. H. O. & Hepatitis, B. vaccines: WHO position paper—July 2017; 27, 369–392. Available at http://www.who.int/wer

[24] Are booster immunizations. Needed for lifelong hepatitis B immunity? European consensus group on hepatitis B immunity. *Lancet* **355** (9203), 561–565 (2000). 10683019

[25] Chaudhari, S., Bhagat, C., Shah, T. & Misra, S. Antibody to hepatitis B surface antigen in vaccinated health care workers. *MJAFI* **64** (4), 329–332 (2008). 10.1016/S0377-1237(08)80013-5 PMC5035263 27688569

[26] El Bahnasy, R. E., Abu Salem, M. E., El Shazly, H. M. & Morad, W. S. Predictors of poor response to the hepatitis B vaccine among healthcare workers at the National liver Institute hospital. *Menoufia Med. J.* **29**, 131–135 (2016).

[27] Cocchio, S. et al. Persistence of anti-Hbs after up to 30 years in health care workers vaccinated against hepatitis B virus. *Vaccines* **9**, 323 (2021). 33915763 10.3390/vaccines9040323 PMC8067181

[28] Pavani, K., Srinivas, M. S., Samhitha, M., Vinayaraj, E. V. & Dass, S. Immune response to hepatitis B vaccine in health care workers in tertiary care hospital in South India. *IJ Microbiol. Res.* **2** (4), 210–213 (2015).

[29] Zamani, F., Fallahian, F., Hashemi, F., Shamsaei, Z. & Alavian, S. M. Immune response to hepatitis B vaccine in health care workers. *Saudi J. Kidney Dis. Transpl.* **22**, 179–184 (2011). 21196642

[30] Ocan, M. et al. Antibody levels and protection after hepatitis B vaccine in adult vaccinated healthcare workers in Northern Uganda. *PLoS One*. **21** (17(1), e0262126 (2022). 10.1371/journal.pone.0262126 PMC8782524 35061771

[31] Centers for Disease Control and Prevention. Interpretation of Hepatitis B Serologic Test Results. https://www.cdc.gov/hepatitis/hbv/interpretationOfHepBSerologicResults.html. Last accessed on 7 December 2023.

[32] Zhao, Y. L. et al. Immune persistence 17 to 20 years after primary vaccination with recombination hepatitis B vaccine (CHO) and the effect of booster dose vaccination. *BMC Infect. Dis.* **30** (19(1), 482 (2019). 10.1186/s12879-019-4134-9 PMC6543564 31146699

